# Evaluation of automated malaria diagnosis using the Sysmex XN-30 analyser in a clinical setting

**DOI:** 10.1186/s12936-019-2655-8

**Published:** 2019-01-22

**Authors:** Evashin Pillay, Shanaz Khodaiji, Belinda C. Bezuidenhout, Monwabisi Litshie, Thérèsa L. Coetzer

**Affiliations:** 1 Wits Research Institute for Malaria, Department of Molecular Medicine and Haematology, School of Pathology, Faculty of Health Sciences, University of the Witwatersrand and National Health Laboratory Service, Johannesburg, South Africa; 2 Hematology Department, P. D. Hinduja National Hospital & Medical Research Centre, Mumbai, India; 3 Department of Microbiology, Chris Hani Baragwanath Academic Hospital, National Health Laboratory Service, Johannesburg, South Africa

**Keywords:** *Plasmodium falciparum*, Automated diagnosis, Sysmex XN-30 analyser

## Abstract

**Background:**

Early and accurate diagnosis of malaria is a critical aspect of efforts to control the disease, and several diagnostic tools are available. Microscopic assessment of a peripheral blood smear enables direct visualization of parasites in infected red blood cells and is the clinical diagnostic gold standard. However, it is subjective and requires a high level of skill. Numerous indirect detection methods are in use, but are not ideal since surrogate markers of infection are measured. This study describes the first clinical performance evaluation of the automated Sysmex XN-30 analyser, which utilizes fluorescence flow cytometry to directly detect and quantitate parasite-infected red blood cells.

**Results:**

Residual EDTA blood samples from suspected malaria cases referred for routine diagnosis were analysed on the XN-30. Parasitaemia was reported as a percentage, as well as absolute numbers of infected red blood cells, and scattergrams provided a visual image of the parasitized red blood cell clusters. The results reported by the XN-30 correlated with microscopy and the analyser demonstrated 100% sensitivity and specificity. Measurements were reproducible and storage of samples at room temperature did not affect the parameters. Several *Plasmodium* species were detected, including *Plasmodium falciparum*, *Plasmodium vivax* and *Plasmodium ovale.* The XN-30 also identified the transmissible gametocytes as a separate cluster on the scattergrams. Abnormal red blood cell indices (low haemoglobin and raised reticulocyte counts), haemoglobinopathies and thrombocytopenia did not interfere with the detection of parasites. The XN-30 also generated a concurrent full blood count for each sample.

**Conclusions:**

The novel technology of the Sysmex XN-30 provides a robust, rapid, automated and accurate platform for diagnosing malaria in a clinical setting. The objective enumeration of red blood cells infected with *Plasmodium* species makes it suitable for global use and allows monitoring of the parasite load once therapy has been initiated, thereby providing an early marker of drug resistance. The automated generation of a full blood count for each sample provides an opportunity for detecting unsuspected cases. Asymptomatic carriers can also be identified, which will be useful in blood transfusion centres, and will enable treatment of these individuals to prevent the spread of the disease.

**Electronic supplementary material:**

The online version of this article (10.1186/s12936-019-2655-8) contains supplementary material, which is available to authorized users.

## Background

### The global burden of disease

Malaria remains one of the most important parasitic diseases globally, with more than 3.2 billion people in 91 countries at risk of infection [1]. The clinical and public health impact of the disease is geographically variable, depending on the intensity of transmission and the parasite species involved. The World Health Organization (WHO) 2018 malaria report indicates that worldwide in 2017, there were approximately 219 million cases with an estimated death toll of 435,000. Ninety-three percent of all deaths due to the disease occurred in sub-Saharan Africa, affecting mainly young children and pregnant women [1]. The report also shows that after an unprecedented period of success in global malaria control, the number of cases has increased, and progress has stalled. Of concern is that several countries which carry a disproportionate burden of disease have reported increases in malaria cases. Notably, the ten highest burden African countries documented an estimated 3.5 million more cases in 2017 compared to the previous year [1]. In this regard effort is needed to reverse this trend and every case of malaria prevented and death averted, contributes to the global drive towards elimination.

### The role of currently recommended malaria diagnostic tools

To combat the burden of malaria effectively, early and accurate diagnosis is important. Not only will a correct diagnosis reliably distinguish malaria from other causes of acute febrile illness, but it will also permit specific treatment to be initiated thereby preventing complications and reducing mortality. Unfortunately, clinical diagnosis alone has a very low specificity and is not accurate enough, even with a travel history, as the presenting clinical symptoms and signs of malaria are often non-specific and can mimic other tropical infections [2]. According to the WHO, malaria diagnosis based solely on clinical features often leads to overtreatment and irrational use of anti-malarial agents [3].

Current diagnostic recommendation by the WHO is for all suspected malaria cases to have a parasitological test to avoid presumptive therapy and minimize unnecessary exposure to anti-malarial drugs. The two recommended methods for parasitological confirmation are microscopy for direct detection of the parasite, and immuno-chromatographic rapid diagnostic tests (RDT) for indirect detection based on the presence of malarial antigen or protein [3]. It is noteworthy that assays based on polymerase chain reaction (PCR) and loop-mediated isothermal amplification (LAMP) have demonstrated high sensitivity and specificity for malaria detection, and by comparison have proven that infections can be missed by microscopy and RDTs [4, 5]. However, these methods are not a practical alternative for utilization in many malaria endemic regions because they are laborious and necessitate a high level of skill.

### Microscopy

For more than a century microscopy has served as the preferred diagnostic test for malaria. Thorough review of a well-prepared and well-stained peripheral blood smear by a skilled microscopist remains the clinical gold standard for malaria parasite detection [6]. Not only does it represent a sensitive and highly specific method for diagnosis, but it also allows quantitation of malaria parasitaemia, species classification and identification of the different life cycle stages [3, 7]. In endemic areas, microscopy can also enable the detection of co-infections including other parasites e.g. babesiosis (a tick‐borne parasitic disease that can be confused with malaria); filariasis (seen as motile microfilariae in thick and thin films); and haemoflagellates e.g. *Trypanosoma* and *Leishmania* species. Bacteria may also be detected e.g. in louse‐ and tick‐borne relapsing fevers, spirochetes of *Borrelia* species can be observed between cells; in septicaemia, bacteria can be seen within neutrophils e.g. streptococci and staphylococci; and in bartonellosis, the causative organism can be appreciated on the surface of red blood cells with associated haemolytic anaemia and spherocytosis. In addition, fungi e.g. *Candida albicans* and *Histoplasma capsulatum* can be observed within neutrophils and monocytes, respectively [8]. However, microscopy is not without its challenges and the quality of results is highly variable, as demonstrated by external assessments recently conducted at selected facilities [9]. Accurate diagnosis depends on the quality of the stained peripheral blood smear, the availability and proper functioning of a microscope, the skill and experience of the microscopist, appropriate methods for validating proficiency, and ongoing supervision and training to maintain an acceptable level of expertise. Further challenges include subjective result interpretation, and a labour-intensive and time-consuming test procedure. An automated microscopist would improve malaria diagnosis. To this end, Eshel and colleagues [10] recently evaluated the performance of a novel desktop instrument, the Parasight (Sight Diagnostics, USA), which uses digital fluorescence microscopy and computer vision algorithms for malaria diagnosis. The sensitivity and specificity for parasite detection when compared to PCR, ranged from 99.0–99.3% and 98.9–100% in two study sites, in India and Kenya, respectively. The instrument was also able to identify *Plasmodium falciparum* and *Plasmodium vivax* species correctly in 100% of cases analysed in India, but in Kenya this decreased to 96.1% for *P. falciparum* cases.

### Immuno-chromatographic RDTs

The other recommended parasitological test that has gained interest in the last two to three decades is the RDT. This technology utilizes monoclonal antibodies on a test strip, which are directed against parasite-specific enzymes or antigens present in the blood of infected individuals [11]. These commercially available tests can detect different malaria species and are generally utilized where quality-assured microscopy is not available [3]. Although they may serve as a useful complement or alternative to microscopy, there are some noteworthy drawbacks. Firstly, they are not quantitative, thereby making them unsuitable for prognostic significance of infection or monitoring of parasite clearance as a measure of treatment efficacy. In addition, they do not distinguish a new infection from a recently treated one, since the malaria antigen can persist for up to 2 months post-treatment [12]. Moreover, they have limited shelf life and are sensitive to high temperatures [7]. They also generate false positive results following cross-reactivity with antigens such as rheumatoid factor [13]. Furthermore, histidine-rich protein 2 (HRP2) gene deletions enable the *P. falciparum* parasite to avoid detection by HRP2-based RDTs, thereby generating false negative results [14]. Lastly, currently available RDTs were developed prior to the discovery that *Plasmodium knowlesi* can cause significant disease in humans and were, therefore, not adequately evaluated for detecting this species [15]. Infection with *P. knowlesi* may thus be missed in patients with a negative RDT if microscopy is not performed, with potentially fatal consequences.

### Malaria detection by automated analysers

As the field of medical diagnostics continues to evolve, there is a constant search for alternative methods to detect and quantify malaria parasites. To reduce analytical time and improve accuracy, automation of the malaria diagnostic process is highly desirable. Automated haematology analysers can offer fast, sensitive and cost-effective assessment of all suspected malaria cases [16]. Several studies have identified unusual light scatter patterns generated during routine full blood count (FBC) analysis of malaria-infected blood samples [17–20]. It has been proposed that these atypical scattergrams are due to white blood cells (WBC), which scatter light differently after they have phagocytosed the parasite and contain birefringent haemozoin pigment [17, 21, 22]. Such evidence is diagnostically impressive; however, these findings only suggest the diagnosis and confirmation by microscopic assessment of a peripheral blood smear will still be mandatory [16]. Also, intra-erythrocytic haemozoin has been detected using specialized flow cytometry methods, however, it is not optimal for diagnosis of *P. falciparum* malaria since the circulating ring forms of the parasite contain minimal amounts of haemozoin [23].

Sysmex haematology analysers (Sysmex Corporation, Kobe, Japan) uniquely utilize the principle of fluorescence flow cytometry to count and differentiate blood cells. Using this technology, the corporation developed a prototype analyser, the XN-10 (M), for the automated detection of malaria. Performance data from the prototype study were used to optimize the software algorithms, which led to the development of the XN-30. This analyser is currently only available for research purposes, but regulatory approval is pending, and the analyser is scheduled for commercial launch in 2019.

A recent evaluation study of the XN-30 by Tougan and colleagues [24] using *P. falciparum* parasites cultured in vitro, demonstrated excellent correlation of automated parasitaemia with microscopy. However, the parasitaemia in healthy asexual *P. falciparum* cultures typically does not exceed 10%, which is much lower than the parasite burden that may be present in severely ill malaria patients. In addition, asexual parasite cultures contain all the intra-erythrocytic developmental stages including ring forms, trophozoites and schizonts, but lack gametocytes, which are formed when a developmental switch to sexual reproduction occurs in selected infected red blood cells (RBC). Parasite cultures therefore differ from the clinical scenario where a blood sample from a patient with *P. falciparum* malaria contains only ring forms and occasionally gametocytes, thus necessitating the evaluation of the analyser in a clinical setting.

This article outlines the technology behind automated malaria detection by Sysmex analysers and report on the initial prototype studies and subsequent evaluation of the diagnostic performance of the XN-30 using blood samples from patients with malaria.

## Methods

The prototype, XN-10 (M), and its successor, the XN-30, utilize the same malaria detection and measurement principle.

### Sysmex malaria detection and measurement principle

Using fluorescence flow cytometry, the XN-30 detects and counts malaria-infected RBCs (MI-RBC) and WBCs. It contains a blue semiconductor laser with a 405 nm beam, making it different from conventional Sysmex haematology analysers, which have a red semiconductor laser with a 633 nm beam. Compared to red light, the wavelength of blue light is shorter, thereby permitting detection of smaller particles. In the process of malaria detection, a newly developed reagent (Lysercell M) partially permeabilizes the cell membrane of RBCs allowing entry of a reagent with fluorescent properties (Fluorocell M), which stains parasite nucleic acids. When a sample is analysed, the RBC count is measured using a sheath flow direct current (DC) detection method and the corresponding malaria-infected RBC count (MI-RBC#; reported as parasites/µL) is measured by fluorescence flow cytometry. The percentage of malaria-infected RBCs (MI-RBC%) is calculated from the ratio of the different RBC counts (MI-RBC# and RBC) obtained from the two methods. Similar to conventional Sysmex analysers, the XN-30 also utilizes the sheath flow DC detection method to determine other indices including the haematocrit and platelet count, and a sodium lauryl sulphate (SLS) method for measuring haemoglobin.

### Sysmex malaria scattergrams

**Fig. 1:**
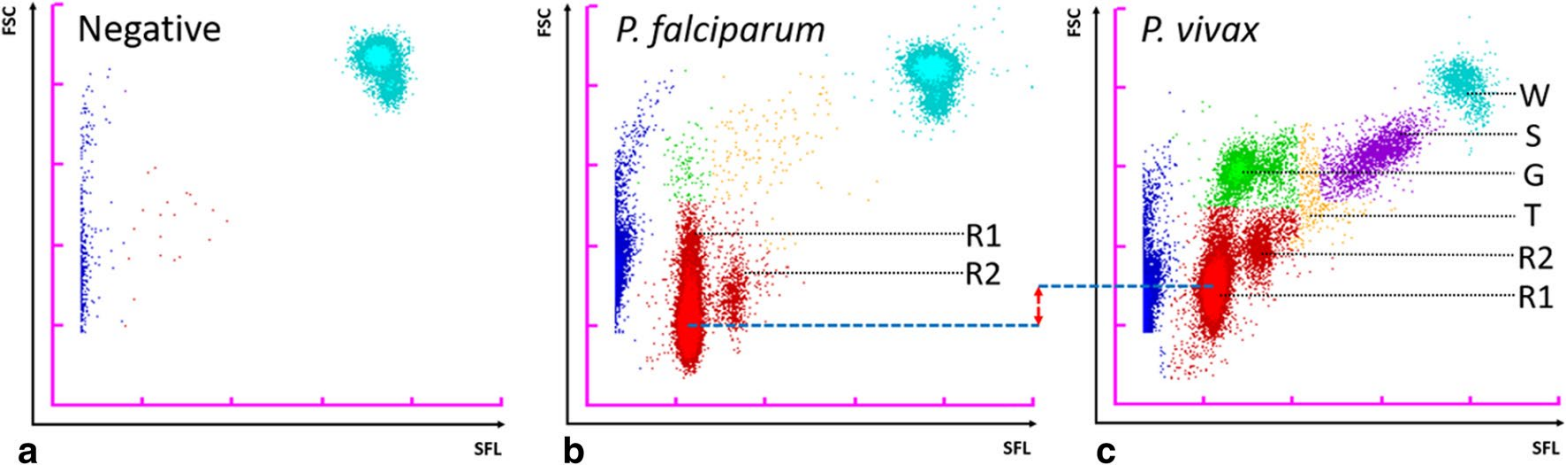
M scattergrams illustrating the principle of detection of red blood cells infected with malaria parasites. SFL: side fluorescence light; FSC: forward scattered light. Samples were analysed in WB mode. **a** Malaria-negative blood sample. Blue dots: non-infected RBCs, platelets and debris; red dots: background noise below the limit of quantitation. **b** RBCs infected with *P. falciparum* (MI-RBCs) showing R1: RBCs infected with 1 ring form, and R2: RBCs infected with 2 ring forms. **c** RBCs infected with *P. vivax* showing different parasite developmental stages. T: trophozoites; G: gametocytes; S: schizonts; W: white blood cells

A malaria (M) scattergram is generated for each sample and consists of side fluorescence light (SFL) intensity on the horizontal axis (x-axis) and forward scattered light (FSC) intensity on the vertical axis (y-axis; Fig. 1). By virtue of the larger size and higher nucleic acid content of WBCs compared to RBCs, they generate a cluster on the upper right corner of the M scattergram (turquoise dots; Fig. 1a). Non-infected RBCs, platelets and debris on the other hand feature on the left side of the M scattergram, along the vertical axis (blue dots; Fig. 1a). The amount of nucleic acid in a single malaria parasite is less than 1/100 of a human WBC [25, 26] and by combining this property with the principle of staining parasite nucleic acids, MI-RBCs can be distinguished from WBCs, non-infected RBCs, platelets and debris (Fig. 1b, c).

The number of parasites in RBCs can also be discriminated by comparing the fluorescence intensity within a sample. The XN-30 can distinguish singly-infected RBCs from those containing two or more ring forms, since the amount of nucleic acid contained within an infected RBC is different, and this is represented on the M scattergram as separate clusters (Fig. 1b, c).

*Plasmodium* develop sequentially through ring forms, trophozoites and schizonts during the asexual life cycle while gametocytes are formed after a developmental trigger to sexual reproduction. Each developmental stage contains different amounts of total stainable nucleic acid. Additionally, MI-RBCs increase in size as the parasite matures from a ring form to a trophozoite and schizont. Conceptually, the intensity of both SFL and FSC will be unique for each of these developmental forms. Thus, the XN-30 categorizes malaria infection within a sample by life cycle stages based on the SFL, reflecting the amount of nucleic acid, and the FSC, reflecting the size of MI-RBCs (Fig. 1b, c). Specifically, each dot on the M scattergram represents the x (SFL): y (FSC) co-ordinates of each individual MI-RBC. The XN-30 software algorithm detects distinct clusters of MI-RBCs within a specified signal range then classifies the life cycle stages and depicts this prediction by assigning specific colours to the dots (Fig. 1b, c). This concept of differentiation is similar to the Adaptive Cluster Analysis System (ACAS), which is utilized by Sysmex X-Class and XN-series haematology analysers for WBC differentiation. In brief, in contrast to fixed gating, the software confirms that a cluster exists (identified by a centroid) and that in the case of ring forms and gametocytes, such clusters are separated by a trough, within a defined signal range, allowing for biological variation. The distinction between trophozoites and schizonts is less clear, as these stages form a continuum (based on size and nucleic acid content), and hence the cut-off for classification between these two stages is fixed. The primary intention of classifying such life cycle stages is not to provide a quantitative differentiation, but rather to show that developmental stages beyond ring forms are present in the peripheral circulation of an infected individual. Evidence of the latter, together with the size of MI-RBCs is used by the XN-30 to differentiate between species (Fig. 1b, c). The RBCs infected with *P. falciparum* contain small ring forms compared to *P. vivax*, which have different physical properties [27], thus generating distinct light scatter patterns. Furthermore, in clinical samples of *P. falciparum* infection, asexual ring forms are predominantly seen, in contrast to *P. vivax* where all the asexual stages are present (Fig. 1b, c).

### XN-30 software improvements

Based on the data analysis of the prototype study, changes were made to the software to improve the limit of quantitation, specificity, and to minimize interferences. Details of the software modification, including the algorithm flow, are provided in Additional file 1: Figs. S1 and S2.

### Study sites

The concept of automated malaria parasite detection using the prototype XN-10 (M) analyser was initially assessed in an urban clinical setting in Johannesburg, in the Republic of South Africa (RSA), from January to June 2014. A second study site, located in Mumbai, India, was added to enrich the data set with *P. vivax* cases from November 2014 to January 2015 and to evaluate the ability of the analyser to differentiate between *Plasmodium* species. The clinical evaluation of the diagnostic performance of the XN-30 was conducted in RSA from January to June 2017.

### Patient samples

In RSA, all adult patients presenting with clinical suspicion of malaria either to Chris Hani Baragwanath Academic Hospital or Charlotte Maxeke Johannesburg Academic Hospital were considered for inclusion. In India, only samples with a microscopic diagnosis of malaria, confirmed at the P. D. Hinduja National Hospital laboratory, were included. At both sites residual blood from specimens collected in EDTA tubes [Vacutainer^®^ K_2_EDTA (1.8 mg/mL), BD Bioscience, USA] and submitted to the local hospital laboratory for routine malaria diagnostic testing were utilized. To be eligible for the study, all samples were required to be at least 1 mL in volume and processed on the analyser by a dedicated operator within 24 h of collection from the patient. This study was approved by the Human Research Ethics Committee of the University of the Witwatersrand (M140995 and M160549) and by the National Health and Education Society (872-14-SK/KS), in RSA and India respectively.

### Evaluation of malaria parasites

#### Sysmex analysers

Samples were analysed on the prototype, XN-10 (M), and the XN-30 analysers in whole blood (WB) mode. In addition, samples were processed in low malaria (LM) and pre-dilute (PD) modes on the XN-30. In the LM mode the volume of each sample measured is triple (180 µL) that of the WB mode (60 µL), to obtain improved sensitivity. In contrast, the PD mode requires only 20 µL of WB sample diluted in 120 µL of a standard diluent (Cellpack DCL), to enable analysis of small blood volume samples such as in elderly and paediatric patients. The XN-30 is equipped to perform automatic conversion of results using the appropriate dilution factor.

Both analysers generate results automatically, which are presented as malaria-negative or malaria-positive, accompanied by a conventional FBC, MI-RBC#, MI-RBC%, an M scattergram and a flag with a suggested species classification, either as suspected *P. falciparum* or suspected ‘others’ (i.e. non-*falciparum* species).

#### Peripheral blood smear microscopy

For the prototype study, thin peripheral blood smears were prepared and evaluated by routine laboratory staff according to standard procedures [28]. For the XN-30 study, the microscopically confirmed parasite density of all malaria-positive samples was correlated with that determined by the analyser to evaluate its performance. For each sample referred for suspected malaria, two thin peripheral blood smears were prepared and stained using the RapiDiff stain kit (Clinical Sciences Diagnostics CC, RSA). All slides were examined by two expert microscopists using the Olympus BX41TF light microscope (Olympus Corporation, Tokyo, Japan) at 500× magnification. The density of malaria parasites was determined according to the current WHO guidelines [28]. Briefly, percentage parasitaemia was calculated after counting the number of MI-RBCs per 10,000 RBCs. If parasites were not visualized after counting 10,000 RBCs, the sample was deemed to be microscopy negative.

#### Immuno-chromatographic RDTs

All samples were tested for the presence of malarial antigens and results recorded as either positive or negative. For the prototype study, the MAKROmed malaria rapid test kit (Makromed Manufacturing (Pty) Ltd, RSA) which detects the *P. falciparum*-specific HRP2 antigen, and the SureTest MAL malaria antigen test kit (pLDH VIVAX + HRP-II FAL; MicroGene Diagnostic Systems (P) Ltd, India), which detects both *P. falciparum* and *P. vivax* species were used. For the XN-30 study, the ICT Malaria dual test kit (ICT diagnostics, RSA), which detects *P. falciparum, P. vivax, Plasmodium malariae* and *Plasmodium ovale* antigens, was employed.

#### Polymerase chain reaction

In RSA, any discordant results between the analysers and other methods (microscopy and/or RDT) were resolved with qualitative PCR assays using *Plasmodium*-specific primers according to a standardized protocol adapted from Hang et al. [29]. For the XN-30 study, the PCR testing was conducted at the National Institute for Communicable Diseases (NICD) in Johannesburg.

### Interference by non-malarial factors

To exclude potential interference by non-malarial factors, several samples, confirmed by microscopy and RDT to be malaria-negative, were processed in WB mode on both analysers and additionally in LM mode on the XN-30. These comprised random routine specimens with isolated low haemoglobin < 10 g/dL, isolated low platelet count < 100 × 10^9^/L and raised reticulocyte counts > 5% or equivalent to > 100,000 × 10^6^/L. Additionally in the prototype study, samples from patients with HIV infection (RSA), confirmed acute Dengue infection and an eosinophil count > 1 × 10^9^/L (India) were included. For the performance evaluation of the XN-30, samples from patients with known haemoglobinopathies (thalassaemia and sickle cell disease) were also analysed.

### Analytical evaluation of the Sysmex XN-30

The XN-30 was assessed for the following additional performance parameters: limit of blank (LoB); limits of detection (LoD) and quantitation (LoQ); carryover; precision and stability.

#### Limit of blank

To determine the LoB, samples confirmed by microscopy and RDT to be malaria-negative, were processed. These comprised random routine specimens with normal FBC results. The LoB was calculated using the formula LoB = Mean_Blank_ + 1.645 (SD_Blank_) [30].

#### Limits of detection and quantitation

To determine the LoD and LoQ, malaria-positive samples, confirmed by microscopy and RDT, were analysed and the MI-RBC# confirmed. Each sample was also typed for ABO blood grouping using a standard kit (Rapid Labs, UK) and then diluted using ABO-compatible malaria-negative blood obtained from volunteers in the RSA research laboratory. Standards comprising various concentrations of parasites were generated for determining the LoQ (WB: 30 parasites/μL; LM: 20 parasites/μL; PD: 40 parasites/μL) and LoD (10, 20, 30, 40, 60 and 100 parasites/μL for each mode). Each standard was analysed ten times consecutively and the LoD was calculated using the formula LoD = LoB + 1.645 (SD_Low concentration sample_) [30].

#### Carryover

Carryover from a malaria-positive sample with high parasite density (> 5% parasitaemia) to a malaria-negative sample was assessed by analysing the positive sample three times consecutively followed by three consecutive analyses of the negative sample. These steps were repeated using Cellpack DCL as a blank instead of the negative sample. Carryover was judged to be clinically significant if the first negative sample or the blank sample demonstrated a MI-RBC# result > LoQ.

#### Precision

To assess for precision, malaria-positive samples, confirmed by microscopy and RDT, and representing high (> 5%), medium (1–2%) and low (< 1%) parasite densities were selected, each of which was analysed ten times consecutively.

#### Stability

To assess the impact of sample age on malaria classification, malaria-positive samples, confirmed by microscopy and RDT, were initially analysed within 24 h of collection from the patient. Each sample was divided into two aliquots, which were stored at 4–8 °C and room temperature (20–25 °C), respectively, and subsequently analysed after approximately 48, 72 and 96 h post sample collection. If there was insufficient sample volume to test both temperatures, preference was given to samples stored at room temperature.

### Purification and detection of gametocytes by the Sysmex XN-30

Gametocytes from NF54 *P. falciparum* parasites were induced in vitro and purified as previously reported [31]. Mature stage V gametocytes were isolated after 14 days in culture using NycoPrep™ (Axis-Shield, Norway) density gradient centrifugation and magnetic separation on LS columns in a MidiMACS magnetic system (Miltenyi Biotec, Germany). Gametocytaemia was assessed in a Neubauer chamber. To evaluate gametocyte detection by the XN-30, malaria-negative WB samples (1 mL) were spiked with increasing volumes of purified gametocytes (1 500 stage V gametocytes/µL) and analysed in WB mode.

### Statistical analysis

Correlation between parasitaemia measured by the XN-30 and microscopy was analysed using regression analysis. The coefficient of determination (R^2^), means, standard deviations (SD), and coefficients of variation (CV % = SD÷mean × 100) were calculated using Analyse-it for Microsoft Excel version 5.01 (Microsoft, Redmond, WA, USA). Confidence intervals (CI; 95%) for the sensitivity and specificity data were also determined using this software.

## Results

### Evaluation of the prototype XN-10 (M)

A total of 1028 samples processed on the prototype analyser gave valid results for further analysis. There were 261 confirmed malaria-positive samples (*P. falciparum:* n = 157; *P. vivax:* n = 104), 261 confirmed malaria-negative samples from patients with an acute febrile illness in whom malaria had been suspected, 278 malaria-negative interference samples, 128 samples with normal FBC values and a further 100 randomly selected FBC samples from the routine laboratory in India.

The malaria detection of the XN-10 (M) analyser was evidenced by a sensitivity of 97.7% (95% confidence interval 95.0–98.9%) and a specificity of 98.7% (95% confidence interval 97.5–99.3%). In eleven smear-negative cases the XN-10 (M) detected malaria, all of which were subsequently confirmed by PCR to be true positive results. Furthermore, there was one “isolated low platelet count” interference sample that appeared to have given a false positive result on the XN-10 (M) but was subsequently confirmed on smear review to be an unsuspected case of true malaria. All other interference samples, as well as all normal samples with interpretable results (refer to next paragraph), were correctly identified as malaria-negative. There was no difference in malaria detection performance based on *Plasmodium* species. The analyser correctly identified the species as *P. falciparum* in 98% of cases (95% confidence interval 94.2–99.3%) and non-*falciparum* in 94.8% of cases (95% confidence interval 88.4–97.8%).

The biggest shortcoming observed was the high percentage (14.8%) of indeterminate results. If the particle count in the “M” channel exceeded the lower LoQ, but no distinct cluster was observed in the malaria gating area, then the analyser generated an “abnormal MI-RBC scattergram” flag and did not issue a report on the presence of malaria. Indeterminate results were largely associated with malaria-negative samples (19.4%; 149/767), with only three of the malaria-positive cases (1.15%; 3/261) being affected. Based on these first performance data, the software was improved, resulting in the XN-30 analyser.

### Evaluation of the Sysmex XN-30

#### Detection of malaria parasites

**Fig. 2:**
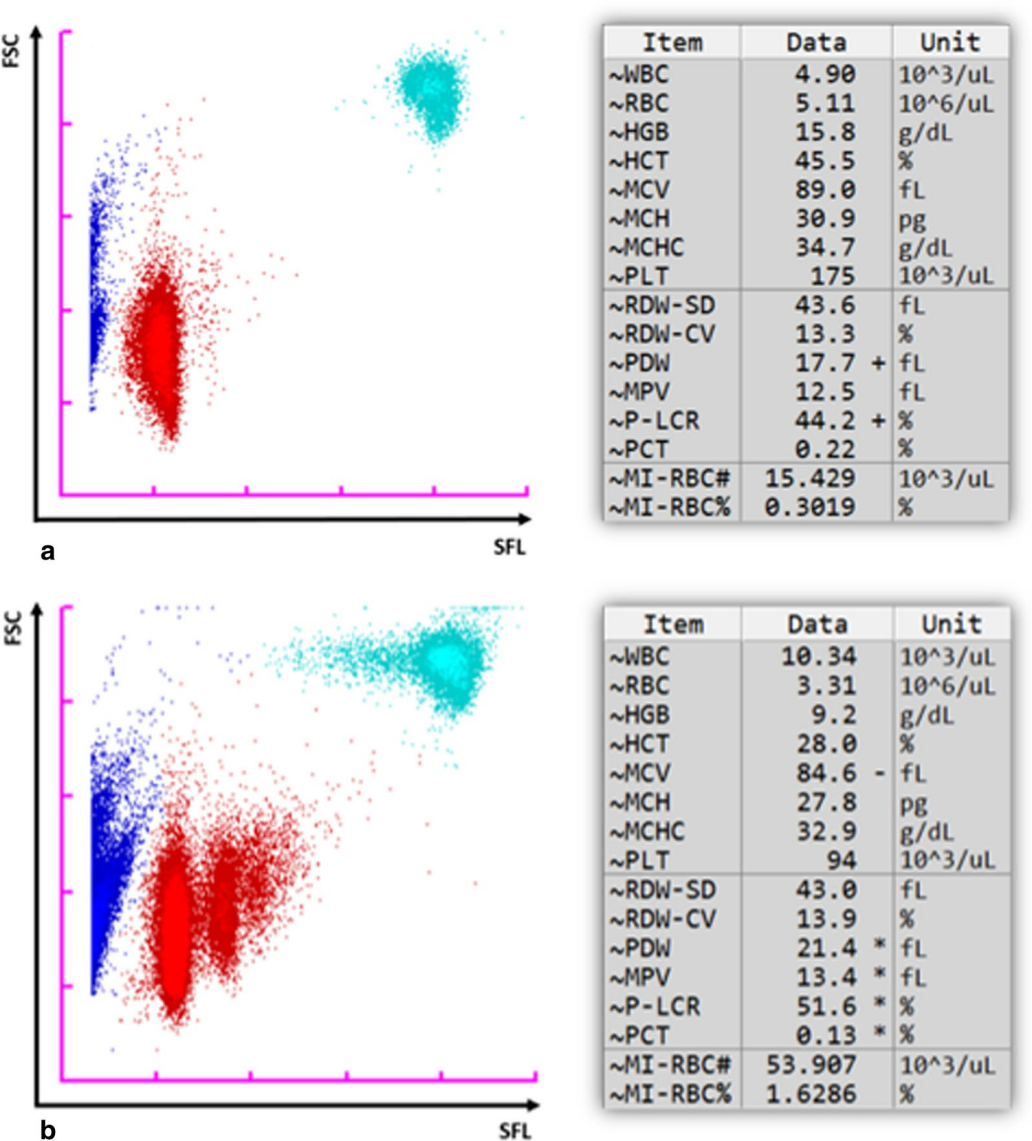
XN-30 M scattergrams illustrating the detection of red blood cells infected with *P. falciparum* malaria parasites (MI-RBCs) using WB mode. The corresponding FBC and quantitative MI-RBC parameters (MI-RBC# and MI-RBC%) are shown to the right of the scattergrams. SFL: side fluorescence light; FSC: forward scattered light; blue dots: non-infected RBCs, platelets and debris; red dots: MI-RBCs; turquoise dots: white blood cells; “+”: value exceeds upper limit; “−”: value exceeds the lower limit; *value has low reliability. **a** Sample with a low parasitaemia of 0.3019%. **b** Sample with a higher parasitaemia of 1.6286%

A total of 191 samples were processed on the XN-30: 124 were confirmed to be malaria-positive (*P. falciparum:* n = 122; *P. ovale:* n = 2) and 67 were confirmed to be malaria-negative by microscopy and RDT, and where necessary also PCR (n = 5). Figure 2 provides examples of M scattergrams of two *P. falciparum* cases. Examples of RBC flags indicating the malaria species as *P. falciparum* ‘P. f’ or ‘others’ are provided in Additional file 1: Fig. S3. The datasets generated and analysed in this study are available in Additional file 2: Tables S1 and S2. Some of the data have been published as an abstract [32].

#### Comparison of parasitaemia between XN-30 analysis and microscopy

**Fig. 3:**
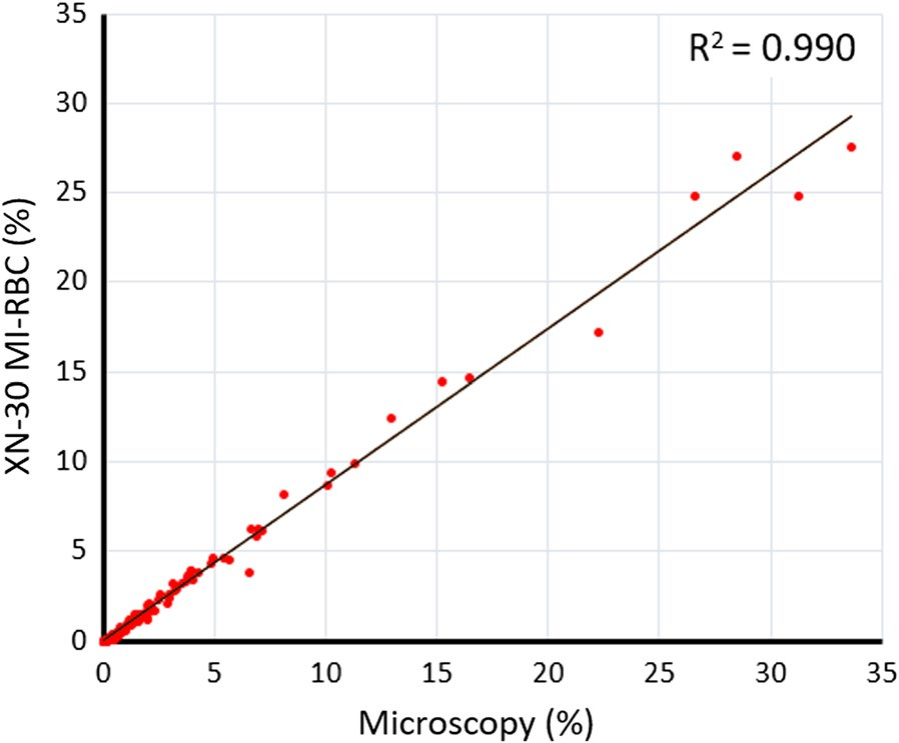
Correlation between *P. falciparum* parasitaemia obtained from the XN-30 (MI-RBC%) and expert microscopy. Samples were analysed in WB mode. R^2^ indicates the coefficient of determination. The diagonal line represents the regression line

To validate the analyser results, parasitaemia determined by the XN-30 was compared to clinical gold standard expert microscopic evaluation. A correlation analysis revealed a strong coefficient of determination with R^2^ = 0.990 for WB mode (Fig. 3). Analysis of samples in LM and PD modes revealed R^2^ values of 0.991 and 0.986, respectively (Additional file 1: Fig. S4). Similar findings were demonstrated by other groups using in vitro *P. falciparum* cultures [24, 33], and suggest that the XN-30 is non-inferior to expert microscopy for the automated quantitation of malaria parasite density.

#### Sensitivity and specificity

The XN-30 demonstrated a sensitivity of 100% (95% confidence interval 97.0–100%) and a specificity of 100% (95% confidence interval 92.6–100%) for malaria parasite detection.

#### Species identification

The LM mode was determined to be most accurate for the correct identification of species as either *P. falciparum* (98% of cases) or “others” (100% of cases).

### Interference by non-malarial factors

**Fig. 4:**
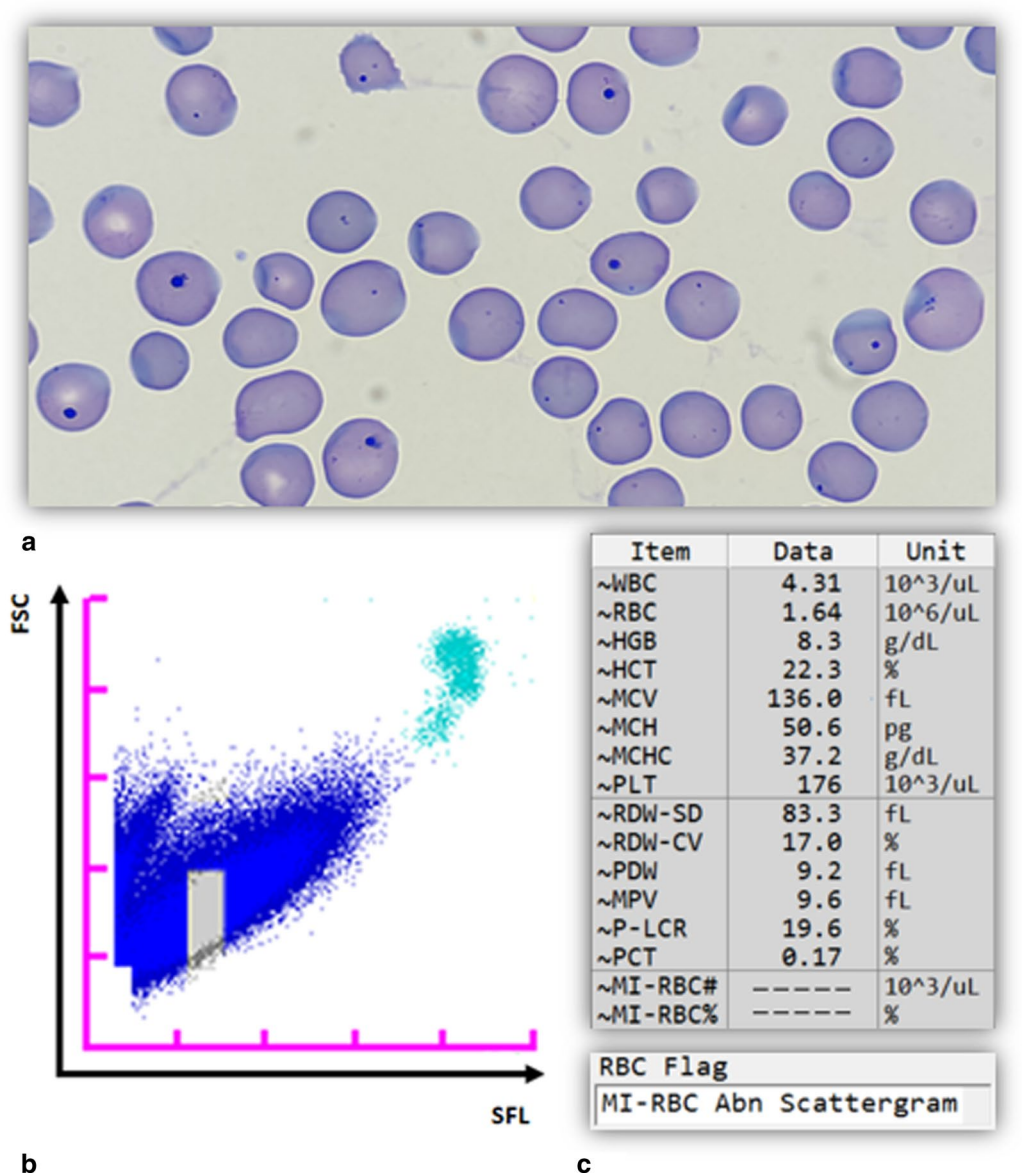
Lack of interference by Howell-Jolly bodies with the quantitative MI-RBC parameters. **a** Peripheral blood smear of the sample with numerous Howell-Jolly body-containing RBCs. **b** The sample was analysed on the XN-30 in WB mode and the M scattergram shows a large blue cluster with a grey area, reflecting the region where MI-RBCs scatter. Turquoise dots: white blood cells. **c** The MI-RBC parameters (MI-RBC# and MI-RBC%) were suppressed and the software triggered an “abnormal MI-RBC scattergram” flag in response to the large blue cluster and the result was reported as indeterminate

To exclude potential interference by non-malarial factors, malaria-negative samples with isolated low haemoglobin levels (n = 22), isolated low platelet counts (n = 24), haemoglobinopathies (n = 23; thalassaemia n = 14 and sickle cell disease n = 9) and raised reticulocyte counts (n = 25) were analysed. Examples of M scattergrams generated from samples of patients with thalassaemia, sickle cell disease and reticulocytosis are shown in Additional file 1: Fig. S5. None of these samples generated a false positive result. However, an “abnormal MI-RBC scattergram” flag occurred in 30% (30/101) and 36% (37/103) of samples analysed in WB and LM modes, respectively, making these results indeterminate. Haemoglobinopathy and reticulocytosis samples were most affected. Notably, during the analysis of samples with raised reticulocyte counts, a single case with numerous Howell-Jolly body-containing RBCs (HJB-RBC) was identified. For this sample, the analyser software triggered an “abnormal MI-RBC scattergram” flag and suppressed the MI-RBC values (Fig. 4). The result was ultimately reported by the XN-30 to be indeterminate, and not falsely positive for malaria, despite the presence of stainable intra-erythrocytic nucleic acids.

### Analytical evaluation of the Sysmex XN-30

#### Detection limits

The LoB (n = 75), LoD (n = 4) and LoQ (n = 3) are shown in Table 1. These findings represent values which are much lower compared to field microscopy, for which the reported LoD is > 100 parasites/µL [34]. The analyser software is designed to suppress any result below the LoQ.

**Table 1:** Limits of blank (LoB), detection (LoD) and quantitation (LoQ) for all modes of the XN-30

	Whole blood (WB)	Low malaria (LM)	Pre-dilute (PD)
Limit of blank (LoB)	13	12	16
Limit of detection (LoD)	22	20	36
Limit of quantitation (LoQ)	30	20	40

Values reported as MI-RBC#

#### Carryover

Carryover was observed in the LM mode in a single instance whereby an MI-RBC# of 21 parasites/μL (LoQ: 20 parasites/μL; Table 1) was reported for a blank measurement, which was immediately preceded by a sample with 5.9% parasitaemia. The manufacturer has since upgraded the XN-30 software to automatically trigger an additional rinse cycle after processing a high (> 5%) parasitaemia sample, to eliminate the risk of any clinically significant carryover. This is currently under evaluation in the Johannesburg research laboratory. Carryover was not observed in the WB or PD modes.

#### Precision

The results of within-run precision analysis for all modes are shown in Additional file 2: Table S3. The CVs of MI-RBC# and MI-RBC% in WB, LM and PD modes ranged from 0.7–3.5%, 0.4–6.7% and 0.4–4.7% respectively.

#### Stability

**Fig. 5:**
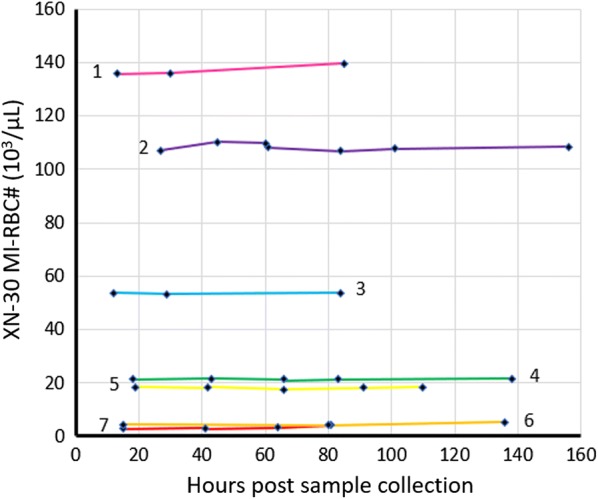
Stability of MI-RBC parameters (MI-RBC#) measured in WB mode on the XN-30. Seven samples with *P. falciparum* parasitaemia ranging from low to high (4–136 × 10^3^/µL), were stored at room temperature and analysed at various time intervals. The first measurement was performed within 24 h of sample collection from the patient and thus the initial point is different for each sample

The MI-RBC# and MI-RBC % parameters were stable when samples were stored at room temperature (Fig. 5) and 4–8 °C (Additional file 1: Fig. S6) for at least 60 h after specimen collection from the patient. The MI-RBC# for the room temperature samples ranged from approximately 4–136 × 10^3^/µL.

### Detection of gametocytes

**Fig. 6:**
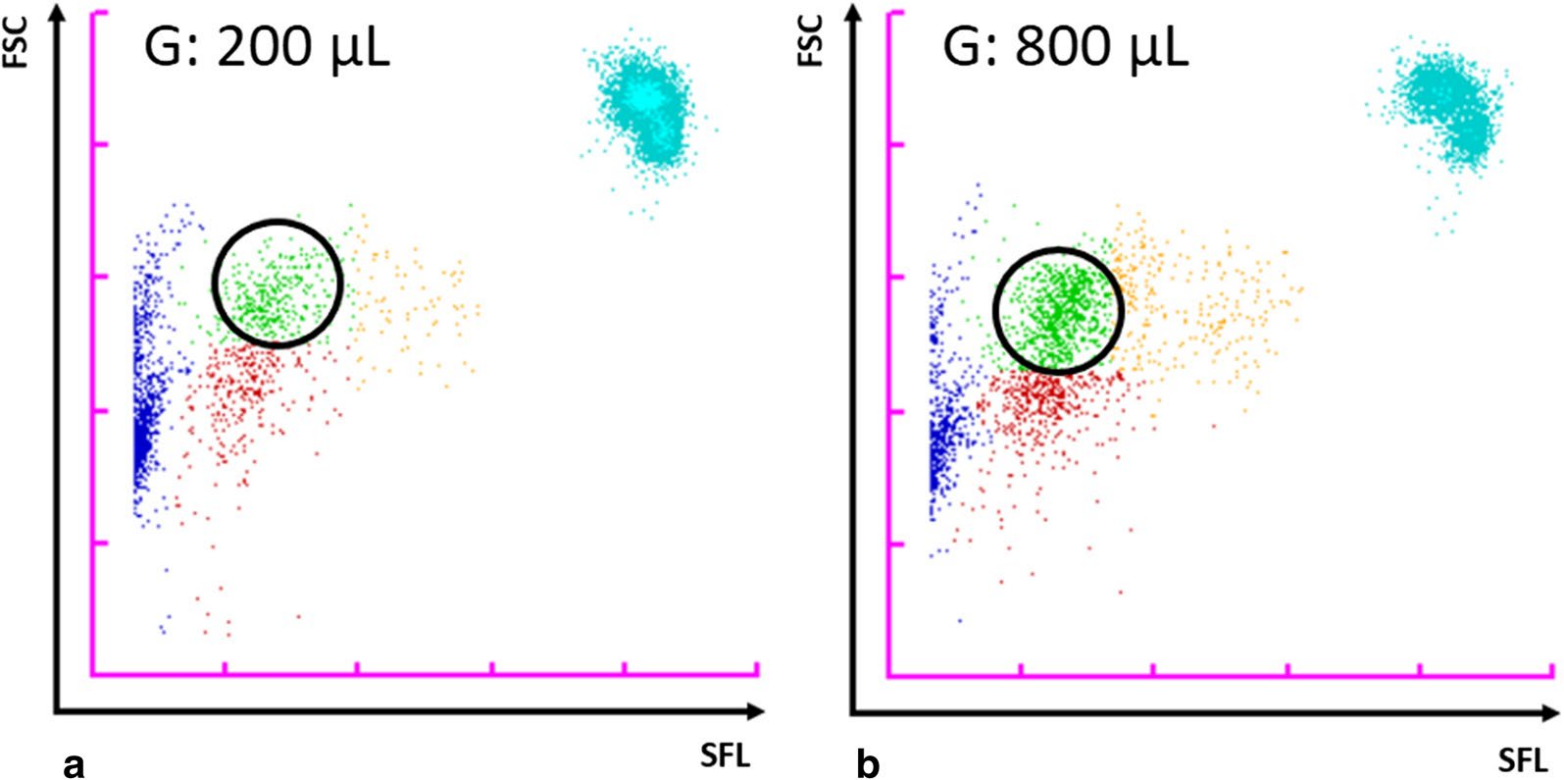
XN-30 M scattergrams illustrating the detection of *P. falciparum* gametocytes. Malaria-negative whole blood (1 mL) spiked with 200 µL (**a**) and 800 µL (**b**) of purified *P. falciparum* gametocytes. SFL: side fluorescence light; FSC: forward scattered light; circled green dots: gametocytes. These samples were analysed in WB mode

The XN-30 generated distinct M scattergrams when *in* *vitro* cultured *P. falciparum* gametocytes were analysed, depicted by discrete clusters (Fig. 6). These findings demonstrate the ability of the analyser to detect the transmissible sexual forms of the parasite. Clinical samples with gametocytes were not available for analysis.

## Discussion

Several groups have described the potential of automated analysers to diagnose malaria based on atypical light scatter patterns due to the presence of haemozoin within phagocytic leukocytes [17, 18, 21]. However, these are indirect methods of detecting the parasite and depend on the host immune response to the pathogen, thus the diagnosis of malaria would still require verification by microscopy. Furthermore, haemozoin can persist in circulating monocytes [35]. Thus, the automated detection of haemozoin suffers the same limitation as RDTs, where positive results may be generated after the patient has been treated with anti-malarial agents, and the parasites have been cleared. Detection of intra-erythrocytic haemozoin has also been reported, but it has low sensitivity for diagnosing *P. falciparum* malaria [23].

This report is the first description of the practical and clinical utility of automated, direct detection of malaria parasites in RBCs from infected patients using the Sysmex XN-30 analyser. It demonstrated excellent sensitivity and specificity for *P. falciparum* parasites, which it quantitated accurately and reproducibly. The parasitaemia results of the XN-30 correlated with the current malaria clinical diagnostic gold standard thereby making it as good as expert microscopy for the diagnosis of malaria in a clinical setting.

This novel technology expands the repertoire of malaria diagnostic tools and provides several advantages, which make it applicable to a wide range of clinical scenarios. The XN-30 detects different life cycle stages of the parasite, based on specific M scattergrams, enabling malaria infections to be categorized into either *P. falciparum* or “others”. Two samples of *P. ovale* were classified as “others” and all *P. falciparum* samples were correctly identified. No mixed infections were observed. Since *P. falciparum* is responsible for > 90% of malaria deaths it is important that this species is correctly identified. In the few cases where both flags were present, this does not pose a diagnostic problem. Clinically relevant species identification is an invaluable property that makes the XN-30 suitable for application on a global scale, particularly in African countries where *P. falciparum* is most prevalent, but also in non-African countries, where other species such as *P. vivax* predominate. Furthermore, it may be of particular value in the detection of *P. knowlesi* for which RDTs are not reliable and microscopy skills may be limited due to the more rural distribution of such cases [15].

The XN-30 is user-friendly, and it takes approximately 1 min to analyse each sample, including specimen aspiration, performance of measurements and reporting of results, which requires minimal technical input or expertise. Likewise, the maintenance requirements, being comprised only of a daily analyser shutdown, are minimal. Furthermore, in the event of any other “as needed” maintenance, the user will be prompted by an on screen “pop-up” message which includes a step by step guide, thereby making all maintenance very simple and easy to execute. In fact, the maintenance of the XN-30 is far simpler than that of the smaller Sysmex haematology analysers, such as the KX-21N, for which there is a large footprint in decentralized laboratories throughout Africa and Asia. Overall, its rapid processing time and objective, automated analysis facilitates high throughput of samples, highlighting the suitability of the XN-30 to malaria endemic regions.

In addition, the MI-RBC parameters are robust and unaffected by room temperature storage of samples, thereby enabling specimens collected even in remote locations to be transported at ambient temperature for analysis.

The quantitative XN-30 technology enables an accurate number of infected RBCs and percentage parasitaemia to be reported, thereby facilitating the monitoring of the decrease in parasite load and thus therapeutic efficacy once treatment with anti-malarial drugs has been initiated. Such properties may allow for early identification of drug resistance to the current front line artemisinin-based combination therapy (ACT), and thus yield valuable data on the spread of artemisinin resistance, which is alarmingly prevalent in the Greater Mekong sub-region [36, 37].

Another important advantage of the XN-30 is that, in addition to the unique property of directly identifying parasites in RBCs, it generates a conventional FBC with each result, which none of the other malaria diagnostic tools offer. This provides clinicians with baseline blood counts and enables them to evaluate the haematological response of the patient during treatment. This feature will also be beneficial in clinical research applications, such as population studies and clinical trials to test new anti-malarial drugs or vaccines. This has recently been exploited in a study of a mouse model of malaria whereby the pharmacokinetics of drugs, as well as therapy-related safety profiles, were investigated on the XN-30 [33]. An additional significant benefit of the concurrent detection of malaria parasites and generation of a FBC is that it may prove useful in identifying unsuspected cases of malaria. In scenarios where there is a low index of suspicion for the disease, a FBC is typically requested as part of an initial diagnostic blood panel in a febrile patient, and the XN-30 will automatically flag the sample if malaria parasites are present. All other diagnostic tools require a directed request from a health care professional to test for malaria. Since prompt diagnosis and initiation of appropriate treatment is vital in preventing complications of malaria and potential death of the patient, the XN-30 may have an impact in identifying infection in countries in the pre-elimination phase and in tourists returning home to non-malaria endemic countries.

Transfusion-transmitted malaria (TTM) is a well-recognized entity and is fast emerging as a major public health concern, particularly in malaria-endemic African countries. The prevalence of *P. falciparum* carriage can range from 2% to more than 55% among healthy blood donors [38–40]. Current screening protocols in these regions pose a diagnostic challenge as the commonly used methods are not sufficiently sensitive [41]. The XN-30 may, therefore, serve as the ideal donor screening tool in blood banks of malaria-endemic regions. The simultaneous haematological evaluation may also provide an indication of donor health status and thus such a strategy may eliminate the risk of TTM and improve the overall quality of blood products obtained.

A final important benefit of the XN-30 is that it can detect gametocytes as demonstrated here and by a Japanese group [24] using in vitro *P. falciparum* culture samples. Gametocytes are critical for parasite survival since they are transmitted to the mosquito vector where they initiate the sexual phase of the parasite life cycle, which results in the production of sporozoites rendering the mosquito infectious and allowing the spread of the disease. The XN-30 may be harnessed to identify asymptomatic carriers who can be treated to prevent transmission of gametocytes and therefore disrupt the parasite life cycle. This strategy is a key component of global initiatives to eliminate the disease. Automated detection of gametocytes may also be useful for further research on gametocytogenesis in patients/asymptomatic carriers and for transmission-blocking drug discovery.

A limitation of the current XN-30 is the number of indeterminate results obtained due to the reported “abnormal MI-RBC scattergram” flags. Despite this only occurring in malaria-negative samples, with no false positive results being generated, it may potentially impact on routine workflow, as affected samples would require microscopic review. Whilst the exact cause of the “abnormal MI-RBC scattergram” flag in the malaria-negative samples is currently unknown, the disproportionate occurrence in specimens obtained from patients with reticulocytosis, suggests that it may be related to high red cell turnover. This is currently under investigation by the manufacturer.

## Conclusions

Timely detection of malaria is life-saving and whilst most health care professionals are trained to recognize the infection, clinical presentation is often non-specific, and diagnosis may initially be overlooked. The XN-30 analyser is an ideal modality for the precise recognition and automated enumeration of malaria parasitaemia. It rapidly detects the actual parasite and not any by-product, such as antigens, phagocytosed parasites or haemozoin, making it more suitable for malaria detection compared to RDTs and other indirect automated methods. Concurrent measurement of haematological parameters is a unique feature that provides valuable data for clinical correlation. Whilst it may not immediately replace microscopy for confirmation of clinical malaria infection, the XN-30 should certainly be considered for utilization as an adjunctive diagnostic tool, because it can facilitate the detection of unsuspected cases. Overall, this technological advance has the potential to significantly impact on malaria diagnostic approaches, therapeutic monitoring and efficacy, anti-malarial drug discovery and clinical trials.

## Additional files

**Additional file 1: Fig. S1.** XN-30 software improvements. **a.** In the prototype software, the M scattergram plot area was divided into 3 regions classifying signals as WBCs (cluster a), non-infected RBCs, platelets and debris (cluster b) and MI-RBCs (cluster c). In brief, the gating strategy sequence was to first identify cluster a and then cluster b. In the prototype, all remaining signals were then assigned to cluster c. Upon further investigation, it was identified that the specified MI-RBC area (cluster c) was too broad, giving rise to false positive MI-RBC results. **b.** The software was subsequently improved for the XN-30 by narrowing the MI-RBC area (cluster c) and incorporating the recognition of one or more distinct clusters (ring forms, gametocytes, trophozoites/schizonts) within an appropriate shape and position as a prerequisite for generating an MI-RBC result. **Fig. S2.** XN-30 malaria classification algorithm. The left side illustrates the algorithm flow if a malaria cluster is not recognized. In such cases, if the MI-RBC# is < LoQ, the sample is reported as malaria negative (MI-RBC green box). However, if the MI-RBC# is ≥ LoQ but signals are detected in the absence of appropriate clustering, or an incorrect cluster shape is generated within the MI-RBC area, then the result is suppressed and an MI-RBC abnormal scattergram flag is generated. An indeterminate result is reported (MI-RBC grey box). An example is presented in M scattergram **(a).** The right side illustrates the algorithm flow if a malaria cluster is recognized. In such cases, if the MI-RBC# is < LoQ, the sample is reported as malaria negative (MI-RBC green box). However, if the MI-RBC# is ≥ LoQ the sample is reported as malaria positive (MI-RBC red box). An example is presented in M scattergram **(b)**. **Fig. S3.** XN-30 M scattergrams, MI-RBC values and the species RBC flags for patient samples infected with **a** *P. falciparum* (P. f) and **b** *P. ovale* (others). Measurements were performed in WB mode. *P. ovale* parasites were confirmed microscopically. SFL: side fluorescence light; FSC: forward scattered light; blue dots: non-infected RBCs, platelets and debris; red dots: MI-RBCs; green and orange dots: different *P. ovale* life cycle forms; turquoise dots: white blood cells. **Fig. S4.** Correlation between *P. falciparum* parasitaemia obtained from the XN-30 (MI-RBC%) and expert microscopy. Samples were analysed in **a** LM mode and **b** PD mode. R^2^ indicates the coefficient of determination. The diagonal lines represent the regression lines. **Fig. S5.** XN-30 M scattergrams of malaria-negative patient samples with **a** thalassaemia, **b** sickle cell disease and **c** reticulocytosis of 13.86%. The grey area within the large blue cluster reflects the region where MI-RBCs scatter, but the software correctly triggered an “abnormal MI-RBC scattergram”. Measurements were performed in WB mode. SFL: side fluorescence light; FSC: forward scattered light; blue dots: non-infected RBCs, platelets and debris; turquoise dots: white blood cells. **Fig. S6.** Stability of MI-RBC parameters (MI-RBC#) at 4–8 °C measured in WB mode on the XN-30. Four samples with *P. falciparum* parasitaemia ranging from **a** high (21–107 × 10^3^/µL), to **b** low (3–4.5 × 10^3^/µL), were stored at 4–8 °C and analysed at various time intervals. The first measurement was performed within 24 h of sample collection from the patient and thus the initial point is different for each sample.

**Additional file 2: Table S1.** WB mode data and M scattergrams. **Table S2.** LM mode data and M scattergrams. **Table S3.** All modes precision data.

## Abbreviations

WHO: World Health Organization
RDT: rapid diagnostic test
PCR: polymerase chain reaction
LAMP: loop-mediated isothermal amplification
HRP2: histidine-rich protein 2
FBC: full blood count
WBC: white blood cell
RBC: red blood cell
DC: direct current
MI-RBC: malaria-infected red blood cell
MI-RBC#: malaria-infected red blood cell count
MI-RBC%: malaria-infected red blood cell percentage
SLS: sodium lauryl sulphate
SFL: side fluorescence light
FSC: forward scattered light
ACAS: adaptive cluster analysis system
RSA: Republic of South Africa
WB: whole blood
LM: low malaria
PD: pre-dilute
NICD: National Institute for Communicable Diseases
HIV: human immunodeficiency virus
LoB: limit of blank
LoD: limit of detection
LoQ: limit of quantitation
SD: standard deviation
CV: coefficient of variation
CI: confidence interval
HJB-RBC: Howell-Jolly body-containing red blood cell
ACT: artemisinin-based combination therapy
TTM: transfusion–transmitted malaria
