# Supplementary figures and images for: Evaluation of automated malaria diagnosis using the Sysmex XN-30 analyser in a clinical setting
Source: Malar J. 2019 Jan 22;18:15. doi: 10.1186/s12936-019-2655-8 (PMC6341646; doi:10.1186/s12936-019-2655-8)

## Slide 1
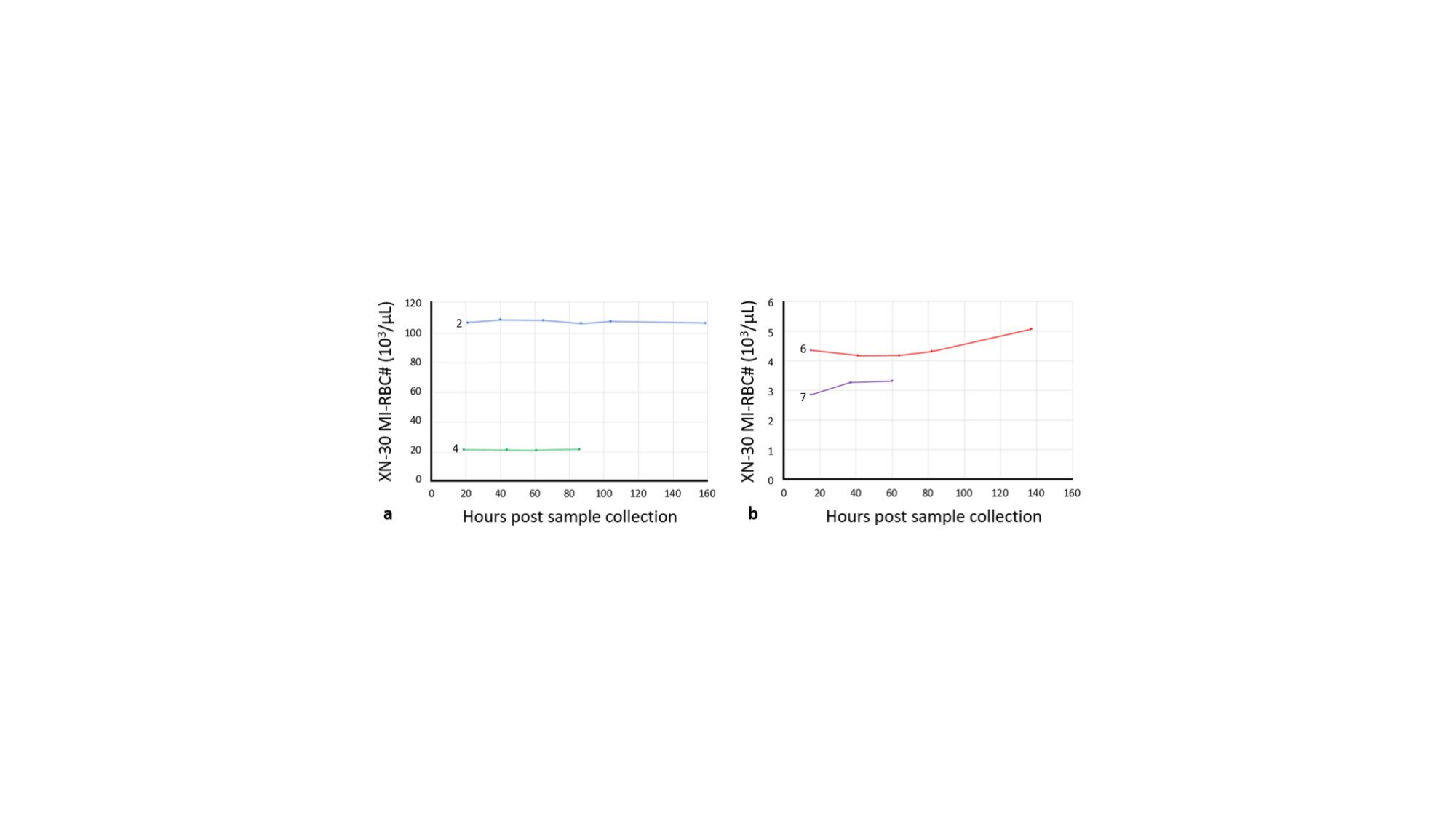

Supplement: Supplementary file 1 — Additional file 1: Fig. S1. XN-30 software improvements. a. In the prototype software, the M scattergram plot area was divided into 3 regions classifying signals as WBCs (cluster a), non-infected RBCs, platelets and debris (cluster b) and MI-RBCs (cluster c). In brief, the gating strategy sequence was to first identify cluster a and then cluster b. In the prototype, all remaining signals were then assigned to cluster c. Upon further investigation, it was identified that the specified MI-RBC area (cluster c) was too broad, giving rise to false positive MI-RBC results. b. The software was subsequently improved for the XN-30 by narrowing the MI-RBC area (cluster c) and incorporating the recognition of one or more distinct clusters (ring forms, gametocytes, trophozoites/schizonts) within an appropriate shape and position as a prerequisite for generating an MI-RBC result. Fig. S2. XN-30 malaria classification algorithm. The left side illustrates the algorithm flow if a malaria cluster is not recognized. In such cases, if the MI-RBC# is < LoQ, the sample is reported as malaria negative (MI-RBC green box). However, if the MI-RBC# is ≥ LoQ but signals are detected in the absence of appropriate clustering, or an incorrect cluster shape is generated within the MI-RBC area, then the result is suppressed and an MI-RBC abnormal scattergram flag is generated. An indeterminate result is reported (MI-RBC grey box). An example is presented in M scattergram (a). The right side illustrates the algorithm flow if a malaria cluster is recognized. In such cases, if the MI-RBC# is < LoQ, the sample is reported as malaria negative (MI-RBC green box). However, if the MI-RBC# is ≥ LoQ the sample is reported as malaria positive (MI-RBC red box). An example is presented in M scattergram (b). Fig. S3. XN-30 M scattergrams, MI-RBC values and the species RBC flags for patient samples infected with a P. falciparum (P. f) and b P. ovale (others). Measurements were performed in WB mode. P. ovale para [file 12936_2019_2655_MOESM1_ESM.zip › Additional file 1, Fig. S6.pptx]

## Slide 1
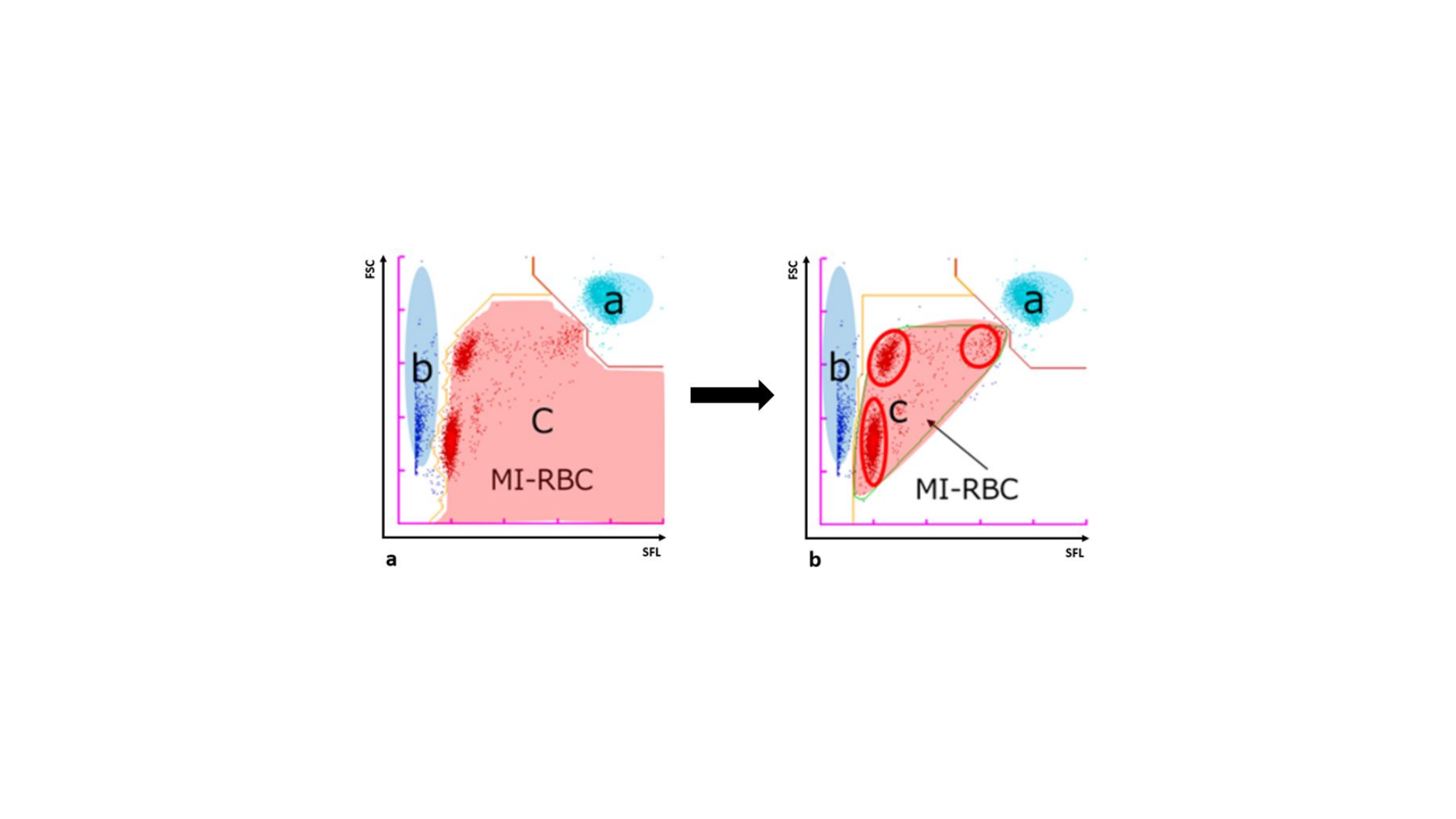

Supplement: Supplementary file 1 — Additional file 1: Fig. S1. XN-30 software improvements. a. In the prototype software, the M scattergram plot area was divided into 3 regions classifying signals as WBCs (cluster a), non-infected RBCs, platelets and debris (cluster b) and MI-RBCs (cluster c). In brief, the gating strategy sequence was to first identify cluster a and then cluster b. In the prototype, all remaining signals were then assigned to cluster c. Upon further investigation, it was identified that the specified MI-RBC area (cluster c) was too broad, giving rise to false positive MI-RBC results. b. The software was subsequently improved for the XN-30 by narrowing the MI-RBC area (cluster c) and incorporating the recognition of one or more distinct clusters (ring forms, gametocytes, trophozoites/schizonts) within an appropriate shape and position as a prerequisite for generating an MI-RBC result. Fig. S2. XN-30 malaria classification algorithm. The left side illustrates the algorithm flow if a malaria cluster is not recognized. In such cases, if the MI-RBC# is < LoQ, the sample is reported as malaria negative (MI-RBC green box). However, if the MI-RBC# is ≥ LoQ but signals are detected in the absence of appropriate clustering, or an incorrect cluster shape is generated within the MI-RBC area, then the result is suppressed and an MI-RBC abnormal scattergram flag is generated. An indeterminate result is reported (MI-RBC grey box). An example is presented in M scattergram (a). The right side illustrates the algorithm flow if a malaria cluster is recognized. In such cases, if the MI-RBC# is < LoQ, the sample is reported as malaria negative (MI-RBC green box). However, if the MI-RBC# is ≥ LoQ the sample is reported as malaria positive (MI-RBC red box). An example is presented in M scattergram (b). Fig. S3. XN-30 M scattergrams, MI-RBC values and the species RBC flags for patient samples infected with a P. falciparum (P. f) and b P. ovale (others). Measurements were performed in WB mode. P. ovale para [file 12936_2019_2655_MOESM1_ESM.zip › Additional file 1, Fig. S1.pptx]

## Slide 1
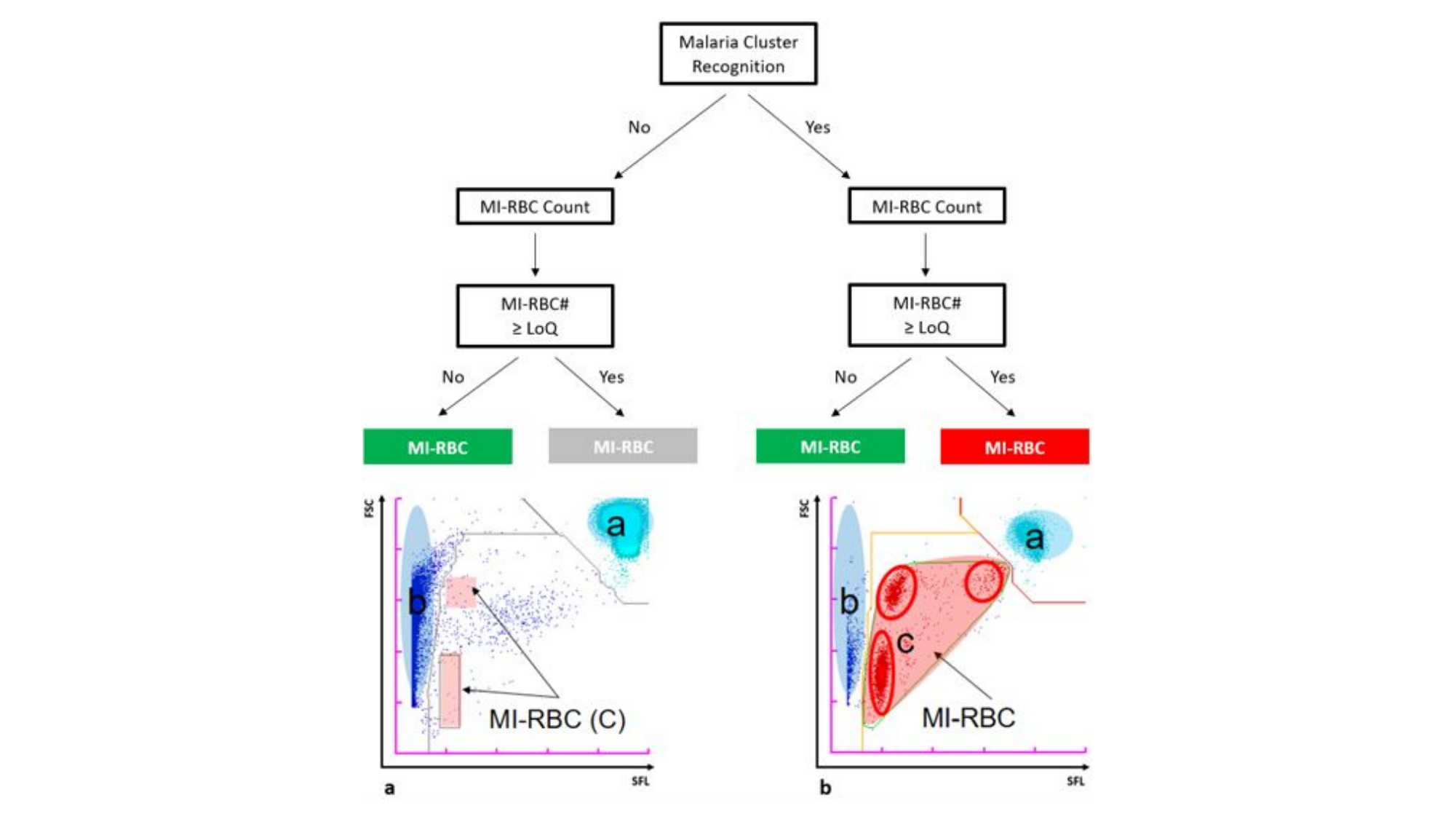

Supplement: Supplementary file 1 — Additional file 1: Fig. S1. XN-30 software improvements. a. In the prototype software, the M scattergram plot area was divided into 3 regions classifying signals as WBCs (cluster a), non-infected RBCs, platelets and debris (cluster b) and MI-RBCs (cluster c). In brief, the gating strategy sequence was to first identify cluster a and then cluster b. In the prototype, all remaining signals were then assigned to cluster c. Upon further investigation, it was identified that the specified MI-RBC area (cluster c) was too broad, giving rise to false positive MI-RBC results. b. The software was subsequently improved for the XN-30 by narrowing the MI-RBC area (cluster c) and incorporating the recognition of one or more distinct clusters (ring forms, gametocytes, trophozoites/schizonts) within an appropriate shape and position as a prerequisite for generating an MI-RBC result. Fig. S2. XN-30 malaria classification algorithm. The left side illustrates the algorithm flow if a malaria cluster is not recognized. In such cases, if the MI-RBC# is < LoQ, the sample is reported as malaria negative (MI-RBC green box). However, if the MI-RBC# is ≥ LoQ but signals are detected in the absence of appropriate clustering, or an incorrect cluster shape is generated within the MI-RBC area, then the result is suppressed and an MI-RBC abnormal scattergram flag is generated. An indeterminate result is reported (MI-RBC grey box). An example is presented in M scattergram (a). The right side illustrates the algorithm flow if a malaria cluster is recognized. In such cases, if the MI-RBC# is < LoQ, the sample is reported as malaria negative (MI-RBC green box). However, if the MI-RBC# is ≥ LoQ the sample is reported as malaria positive (MI-RBC red box). An example is presented in M scattergram (b). Fig. S3. XN-30 M scattergrams, MI-RBC values and the species RBC flags for patient samples infected with a P. falciparum (P. f) and b P. ovale (others). Measurements were performed in WB mode. P. ovale para [file 12936_2019_2655_MOESM1_ESM.zip › Additional file 1, Fig. S2.pptx]

## Slide 1
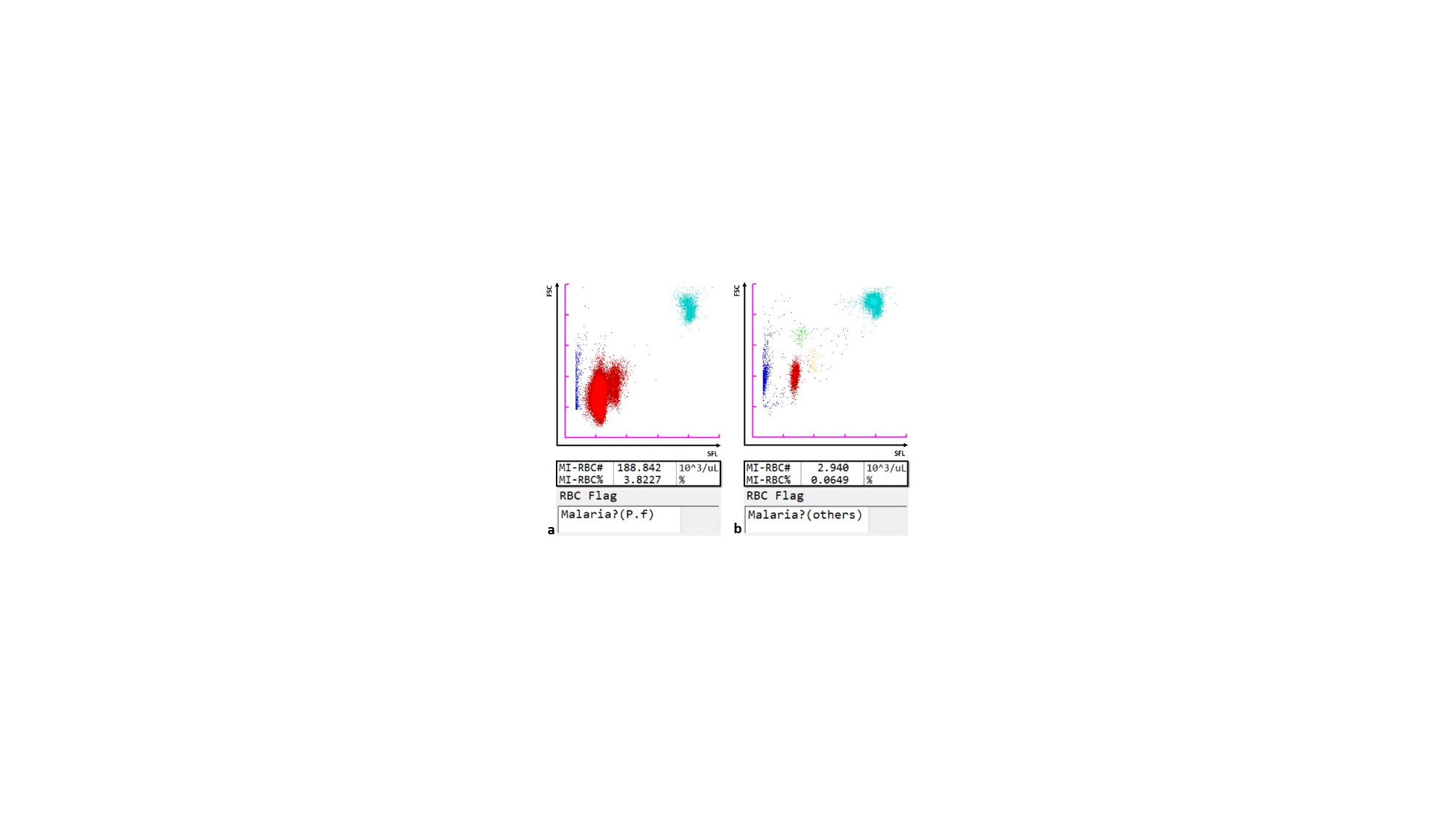

Supplement: Supplementary file 1 — Additional file 1: Fig. S1. XN-30 software improvements. a. In the prototype software, the M scattergram plot area was divided into 3 regions classifying signals as WBCs (cluster a), non-infected RBCs, platelets and debris (cluster b) and MI-RBCs (cluster c). In brief, the gating strategy sequence was to first identify cluster a and then cluster b. In the prototype, all remaining signals were then assigned to cluster c. Upon further investigation, it was identified that the specified MI-RBC area (cluster c) was too broad, giving rise to false positive MI-RBC results. b. The software was subsequently improved for the XN-30 by narrowing the MI-RBC area (cluster c) and incorporating the recognition of one or more distinct clusters (ring forms, gametocytes, trophozoites/schizonts) within an appropriate shape and position as a prerequisite for generating an MI-RBC result. Fig. S2. XN-30 malaria classification algorithm. The left side illustrates the algorithm flow if a malaria cluster is not recognized. In such cases, if the MI-RBC# is < LoQ, the sample is reported as malaria negative (MI-RBC green box). However, if the MI-RBC# is ≥ LoQ but signals are detected in the absence of appropriate clustering, or an incorrect cluster shape is generated within the MI-RBC area, then the result is suppressed and an MI-RBC abnormal scattergram flag is generated. An indeterminate result is reported (MI-RBC grey box). An example is presented in M scattergram (a). The right side illustrates the algorithm flow if a malaria cluster is recognized. In such cases, if the MI-RBC# is < LoQ, the sample is reported as malaria negative (MI-RBC green box). However, if the MI-RBC# is ≥ LoQ the sample is reported as malaria positive (MI-RBC red box). An example is presented in M scattergram (b). Fig. S3. XN-30 M scattergrams, MI-RBC values and the species RBC flags for patient samples infected with a P. falciparum (P. f) and b P. ovale (others). Measurements were performed in WB mode. P. ovale para [file 12936_2019_2655_MOESM1_ESM.zip › Additional file 1, Fig. S3.pptx]

## Slide 1
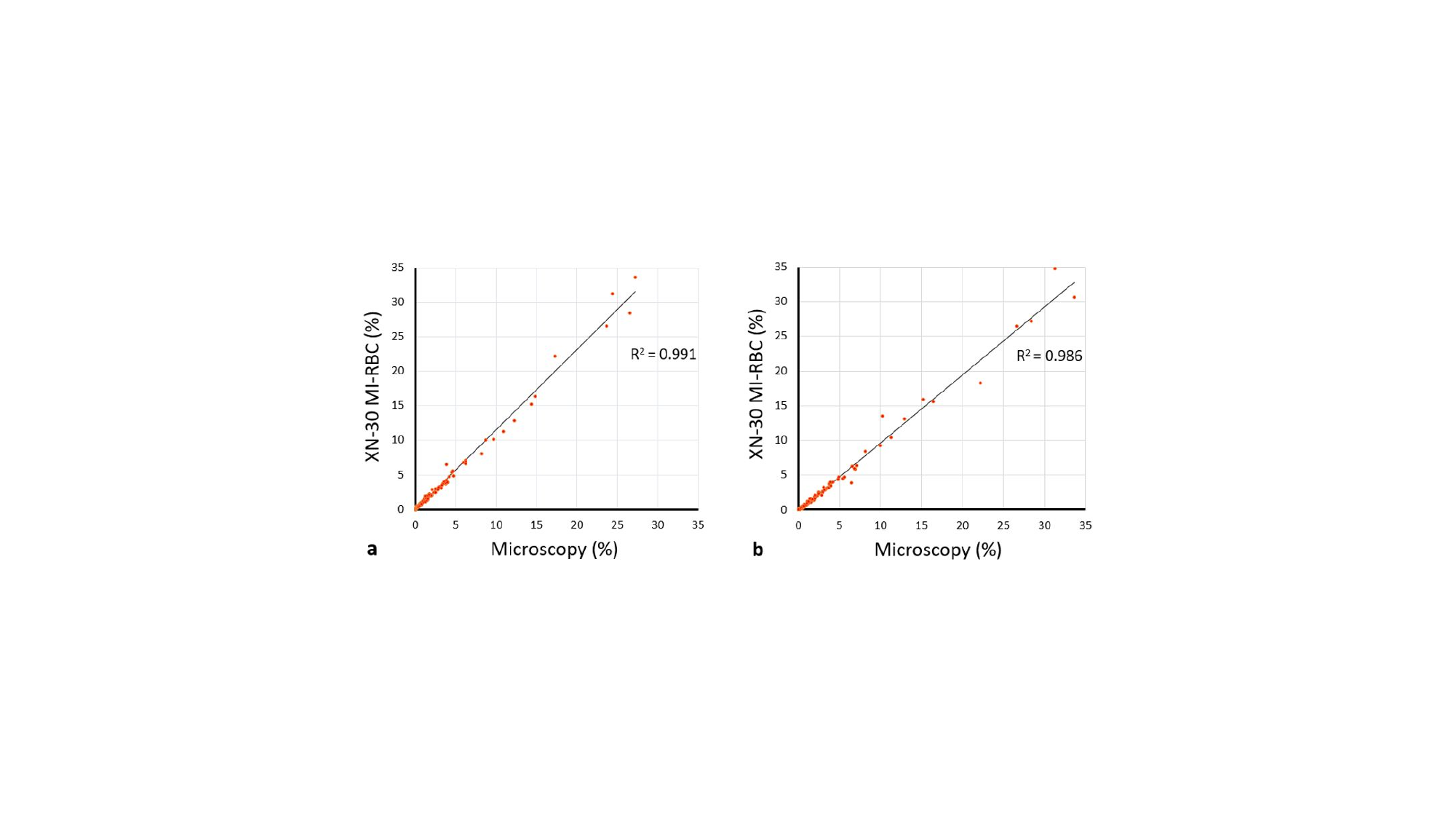

Supplement: Supplementary file 1 — Additional file 1: Fig. S1. XN-30 software improvements. a. In the prototype software, the M scattergram plot area was divided into 3 regions classifying signals as WBCs (cluster a), non-infected RBCs, platelets and debris (cluster b) and MI-RBCs (cluster c). In brief, the gating strategy sequence was to first identify cluster a and then cluster b. In the prototype, all remaining signals were then assigned to cluster c. Upon further investigation, it was identified that the specified MI-RBC area (cluster c) was too broad, giving rise to false positive MI-RBC results. b. The software was subsequently improved for the XN-30 by narrowing the MI-RBC area (cluster c) and incorporating the recognition of one or more distinct clusters (ring forms, gametocytes, trophozoites/schizonts) within an appropriate shape and position as a prerequisite for generating an MI-RBC result. Fig. S2. XN-30 malaria classification algorithm. The left side illustrates the algorithm flow if a malaria cluster is not recognized. In such cases, if the MI-RBC# is < LoQ, the sample is reported as malaria negative (MI-RBC green box). However, if the MI-RBC# is ≥ LoQ but signals are detected in the absence of appropriate clustering, or an incorrect cluster shape is generated within the MI-RBC area, then the result is suppressed and an MI-RBC abnormal scattergram flag is generated. An indeterminate result is reported (MI-RBC grey box). An example is presented in M scattergram (a). The right side illustrates the algorithm flow if a malaria cluster is recognized. In such cases, if the MI-RBC# is < LoQ, the sample is reported as malaria negative (MI-RBC green box). However, if the MI-RBC# is ≥ LoQ the sample is reported as malaria positive (MI-RBC red box). An example is presented in M scattergram (b). Fig. S3. XN-30 M scattergrams, MI-RBC values and the species RBC flags for patient samples infected with a P. falciparum (P. f) and b P. ovale (others). Measurements were performed in WB mode. P. ovale para [file 12936_2019_2655_MOESM1_ESM.zip › Additional file 1, Fig. S4.pptx]

## Slide 1
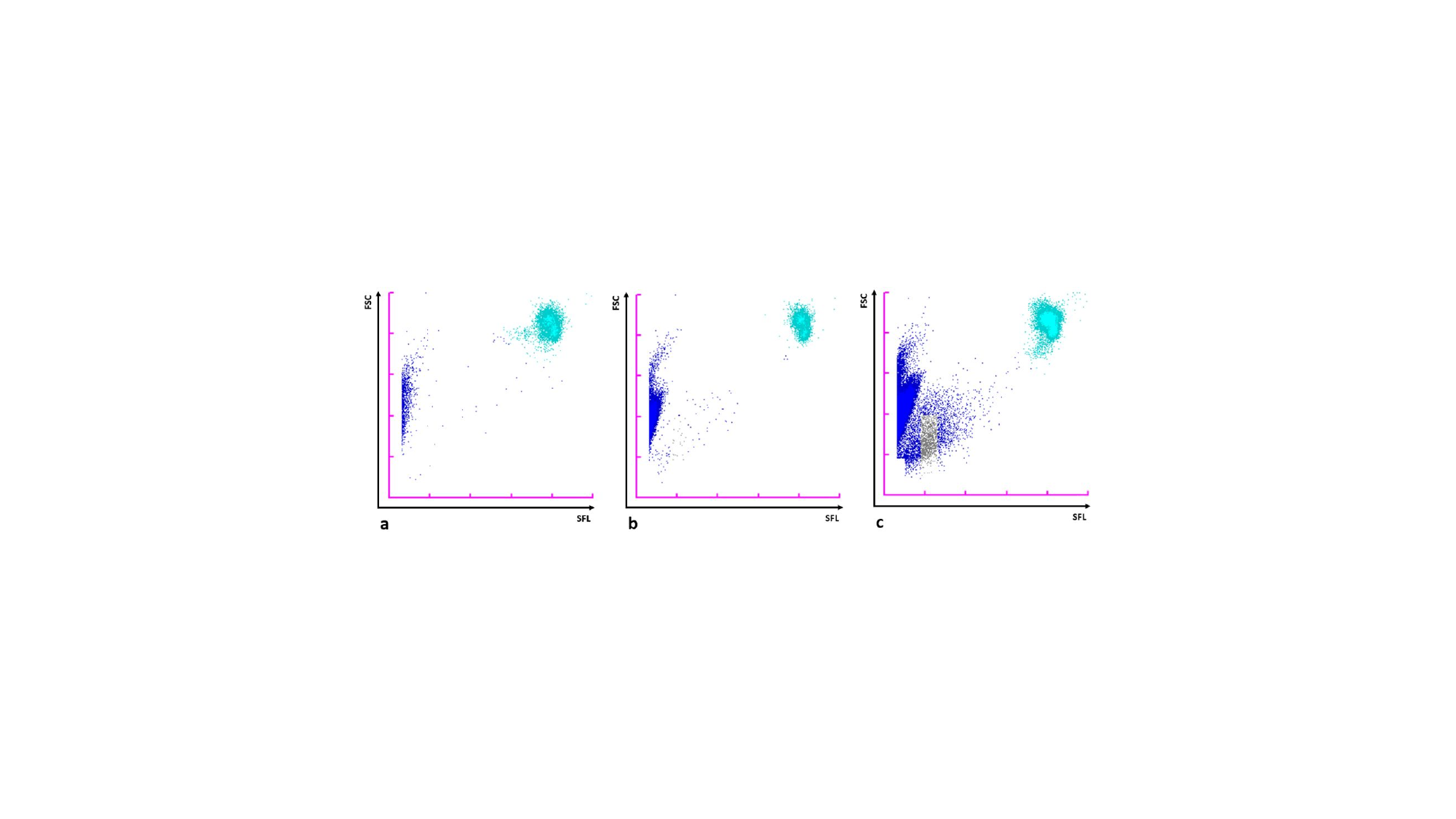

Supplement: Supplementary file 1 — Additional file 1: Fig. S1. XN-30 software improvements. a. In the prototype software, the M scattergram plot area was divided into 3 regions classifying signals as WBCs (cluster a), non-infected RBCs, platelets and debris (cluster b) and MI-RBCs (cluster c). In brief, the gating strategy sequence was to first identify cluster a and then cluster b. In the prototype, all remaining signals were then assigned to cluster c. Upon further investigation, it was identified that the specified MI-RBC area (cluster c) was too broad, giving rise to false positive MI-RBC results. b. The software was subsequently improved for the XN-30 by narrowing the MI-RBC area (cluster c) and incorporating the recognition of one or more distinct clusters (ring forms, gametocytes, trophozoites/schizonts) within an appropriate shape and position as a prerequisite for generating an MI-RBC result. Fig. S2. XN-30 malaria classification algorithm. The left side illustrates the algorithm flow if a malaria cluster is not recognized. In such cases, if the MI-RBC# is < LoQ, the sample is reported as malaria negative (MI-RBC green box). However, if the MI-RBC# is ≥ LoQ but signals are detected in the absence of appropriate clustering, or an incorrect cluster shape is generated within the MI-RBC area, then the result is suppressed and an MI-RBC abnormal scattergram flag is generated. An indeterminate result is reported (MI-RBC grey box). An example is presented in M scattergram (a). The right side illustrates the algorithm flow if a malaria cluster is recognized. In such cases, if the MI-RBC# is < LoQ, the sample is reported as malaria negative (MI-RBC green box). However, if the MI-RBC# is ≥ LoQ the sample is reported as malaria positive (MI-RBC red box). An example is presented in M scattergram (b). Fig. S3. XN-30 M scattergrams, MI-RBC values and the species RBC flags for patient samples infected with a P. falciparum (P. f) and b P. ovale (others). Measurements were performed in WB mode. P. ovale para [file 12936_2019_2655_MOESM1_ESM.zip › Additional file 1, Fig. S5.pptx]
